# Pulse-contour derived cardiac output measurements in morbid obesity: influence of actual, ideal and adjusted bodyweight

**DOI:** 10.1007/s10877-017-0053-8

**Published:** 2017-08-18

**Authors:** Chantal A. Boly, Pieter Schraverus, Floris van Raalten, Jan-Willem Coumou, Christa Boer, Simone van Kralingen

**Affiliations:** 1 Department of Anesthesiology, Institute for Cardiovascular Research, VU University Medical Center, De Boelelaan 1117, 1081 HV Amsterdam, The Netherlands; 2 Department of Anesthesiology, Onze Lieve Vrouwe Gasthuis, Amsterdam, The Netherlands

**Keywords:** Obesity, Bodyweight, Cardiac output, Pulse contour

## Abstract

The non-invasive Nexfin cardiac output (CO) monitor shows a low level of agreement with the gold standard thermodilution method in morbidly obese patients. Here we investigate whether this disagreement is related to excessive bodyweight, and can be improved when bodyweight derivatives are used instead. We performed offline analyses of cardiac output recordings of patient data previously used and partly published in an earlier study by our group. In 30 morbidly obese patients (BMI > 35 kg/m^2^) undergoing laparoscopic gastric bypass, cardiac output was simultaneously determined with PiCCO thermodilution and Nexfin pulse-contour method. We investigated if agreement of Nexfin-derived CO with thermodilution CO improved when ideal and adjusted—instead of actual- bodyweight were used as input to the Nexfin. Bodyweight correlated with the difference between Nexfin-derived and thermodilution-derived CO (r = −0.56; p = 0.001). Bland Altman analysis of agreement between Nexfin and thermodilution-derived CO revealed a bias of 0.4 ± 1.6 with limits of agreement (LOA) from −2.6 to 3.5 L min when actual bodyweight was used. Bias was −0.6 ± 1.4 and LOA ranged from −3.4 to 2.3 L min when ideal bodyweight was used. With adjusted bodyweight, bias improved to 0.04 ± 1.4 with LOA from −2.8 to 2.9 L min. Our study shows that agreement of the Nexfin-derived with invasive CO measurements in morbidly obese patients is influenced by body weight, suggesting that Nexfin CO measurements in patients with a BMI above 35 kg/m^2^ should be interpreted with caution. Using adjusted body weight in the Nexfin CO-trek algorithm reduced the bias.

## Introduction

Morbidly obese patients are a high-risk patient population that may particularly benefit from perioperative cardiac output monitoring due to their increased risk of cardiac and non-cardiac morbidity and mortality when undergoing surgery [1, 2]. In particular, continuous cardiac output monitoring devices such as the CNAP^®^ or Nexfin^®^/Clearsight^®^ may be of particular importance in this population due to their non-invasiveness nature [3–6]. Although morbid obesity is increasingly prevalent and these patients now form an important part of the anesthesia population [7], studies on the applicability of these non-invasive cardiac output measurement devices in this specific population remain limited.

Recently, our group showed an unacceptable agreement and trending capability of cardiac output measured by the Nexfin device when compared to a gold standard thermodilution method in morbidly obese patients undergoing bariatric surgery [8]. The Nexfin calculates cardiac output from non-invasive blood pressure measurements, so-called pulse contour analysis [3]. This CO-trek algorithm calculates stroke volume by dividing the pulsatile systolic area of the arterial pressure waveform by left ventricular input impedance during ejection [3, 9]. Estimation of impedance by the CO-trek algorithm is based on an in vitro study using human aorta samples and requires input of age, gender, height and weight into the algorithm [10]. We however showed that this CO-trek algorithm results in a cardiac output estimation with a low level of agreement with invasive cardiac output measurements in patients with extreme bodyweight [8].

In order to understand these findings we here hypothesize that the disagreement of cardiac output estimations by the Nexfin with thermodilution-based cardiac output measurements is due to the excessive bodyweight of these patients. We presume that the CO-trek algorithm cannot accurately be extrapolated to patients with extreme body weight.

Here we investigate whether the disagreement between the Nexfin-derived cardiac output and the (gold standard) thermodilution method is associated with the excessive bodyweight, and whether this improves when bodyweight derivatives such as ideal and adjusted bodyweight are used instead.

## Methods

In this study we performed offline analyses of cardiac output recordings of a part of the patient data previously used and partly published in the study by Schraverus et al. [8]. The study population consisted of patients with a body mass index over 35 kg/m^2^ scheduled for laparoscopic gastric bypass surgery under general anesthesia between January and December 2014. Approval was obtained from the Human Subjects Committee of the Medical Research Ethics Committees United (MEC-U, Nieuwegein, the Netherlands; NL45442.100.13) and written informed consent was obtained from each participating patient. All patients underwent anesthesia according to a standardized protocol as described in the aforementioned study and were intubated and mechanically ventilated. Subsequently measurements of cardiac output using PiCCO thermodilution and Nexfin were simultaneously performed after induction of anesthesia.

### Cardiac output measurements: thermodilution and Nexfin

Thermodilution cardiac output measurements were performed using the PiCCO 2 system (Pulsion Medical, Germany). The arterial line (4F, 16 cm; Pulsion Medical Systems, Feldkirchen, Germany) was inserted in a brachial artery and a central venous line (ArrowGuard; 7F, 20 cm; Teleflex, Hilversum, the Netherlands) was placed in the right internal jugular vein by ultrasound guidance. The PiCCO was connected to the arterial catheter and to a monitoring probe (PulsionMedical Systems, Feldkirchen, Germany). The monitoring probe measured the moment of injection, temperature and injection pressure of each saline bolus in duplo. A thermodilution measurement from the PiCCO was obtained by injecting 20 ml of cold saline (5–8 °C). A measurement was considered technically valid if the delta temperature was more than 0.2 °C.

Non-invasive cardiac output was measured using the Nexfin^®^ device (Edward Lifesciences, Amsterdam, the Netherlands). An appropriately sized finger cuff was placed on the second phalanx of the hand contralateral to the arm used to place the brachial artery catheter. Patient’s bodyweight, length and age were used as input in the device according to the manufacturer’s instructions. The Nexfin measures blood pressure with a finger cuff using the volume-clamp method of Penaz [11], and finger arterial pressure is then reconstructed into a brachial arterial pressure waveform. Stroke volume and thus cardiac output are then calculated from the arterial pressure waveform [12]. The CO-trek algorithm is based on the Modelflow method which simulates a three-element windkessel model, taking into account aortic impedance, compliance and peripheral resistance [9, 13]. Mathematical functions were derived from early in vitro studies on aortic samples by Langewouters [10], which describe the non-linear relationships of the aorta. A built-in physiological calibration (Physiocal™, BMEYE, Amsterdam, the Netherlands) adjusts the set point of the clamped artery after a maximum of 80 heartbeats [14].

### Re-estimation of Nexfin cardiac output with ideal- and adjusted bodyweight

Ideal bodyweight was calculated with a simple but valid formula as 22 × length (m)^2^ [15] and adjusted bodyweight as IBW + 0.4 (actual bodyweight-IBW). A formula was created from a weight- versus cardiac output curve of one measurement to adjust the cardiac output value as determined with actual bodyweight to cardiac output with ideal and adjusted bodyweight as input.

### Statistical analysis

Statistical data analyses were carried out using a SPSS statistical software package version 19.0 (IBM, New York, NY, USA). Standard descriptive statistics were used to describe the patient characteristics and respiratory data and expressed as mean ± standard deviation (SD), median with interquartile range (IQR) or frequencies. Correlation between bodyweight and the difference between Nexfin and thermodilution-derived cardiac output values was estimated using Pearson’s correlation. Agreement between Nexfin and thermodilution derived cardiac output values with the different weight inputs was determined with Bland Altman plots [16]. A p-value < 0.05 was considered as statistically significant.

## Results

### Patient population

In total, 30 patients were included (20 females and 10 males) who were all morbidly obese with an average mean body mass index of 45 ± 6 kg/m^2^. Mean body weight was 127 ± 21 kg, while mean ideal and adjusted bodyweight estimated 63 ± 7 and 88 ± 11 kg, respectively.

### Difference between Nexfin and thermodilution-derived cardiac output values

**Fig. 1:**
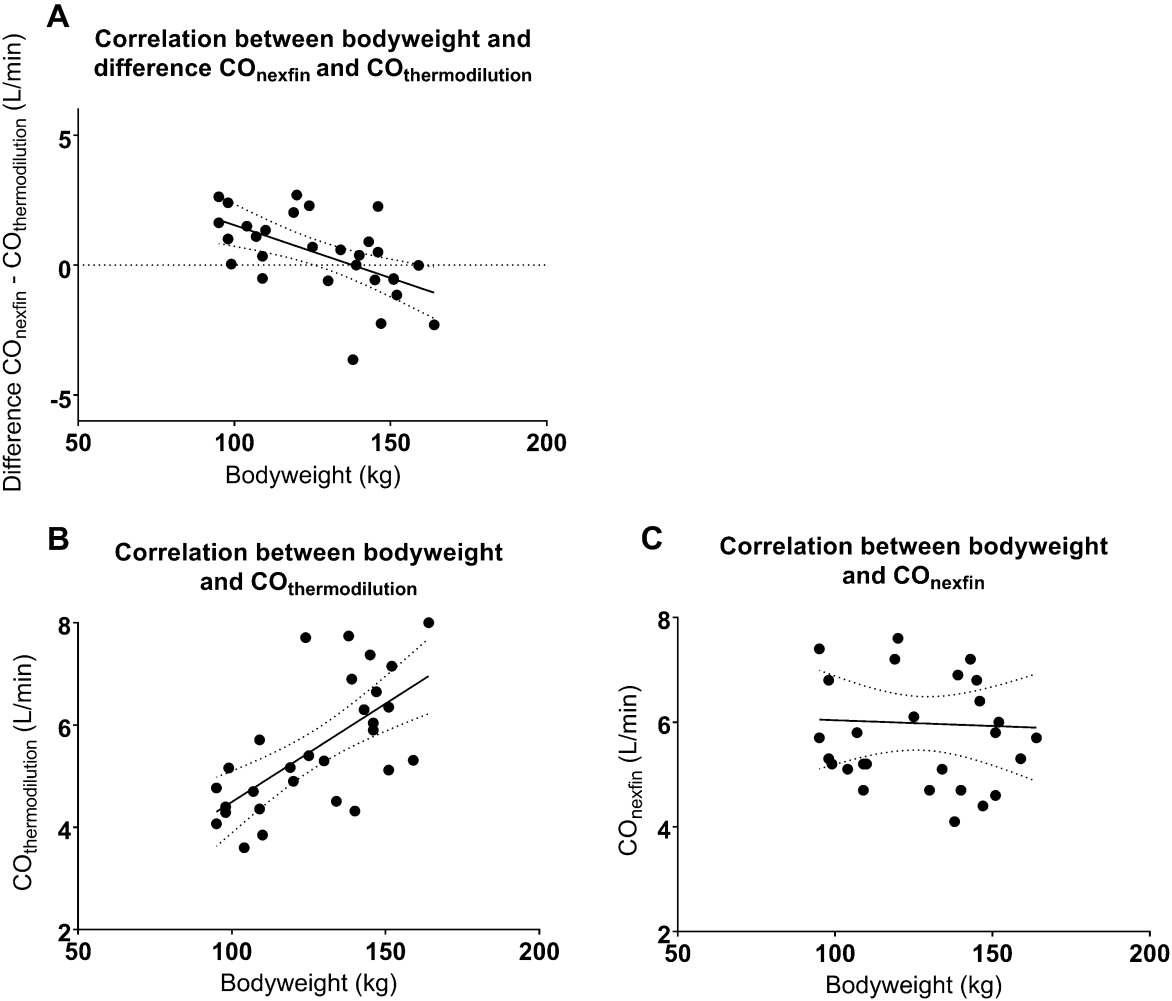
**a** Correlation between bodyweight and difference in CO between methods, Pearson’s r −0.56 (p = 0.001).** b** Correlation between bodyweight and thermodilution cardiac output, Pearson’s r 0.66 (p = 0.0001). **c** Correlation between bodyweight and Nexfin-derived cardiac output, Pearson’s r −0.04 (p = 0.85)

There was a significant correlation between bodyweight and the difference between the Nexfin-derived and thermodilution-derived cardiac output values (r = −0.56; p = 0.001; Fig. 1a). Patients with a higher bodyweight also had higher cardiac output value when determined with thermodilution (r = 0.66 p = 0.0001, Fig. 1b), this correlation did not exist with Nexfin-derived measurements of cardiac output (r = −0.04, p = 0.85, Fig. 1c).

### Agreement between Nexfin- and thermodilution-derived cardiac output with actual, ideal and adjusted bodyweight

**Fig. 2:**
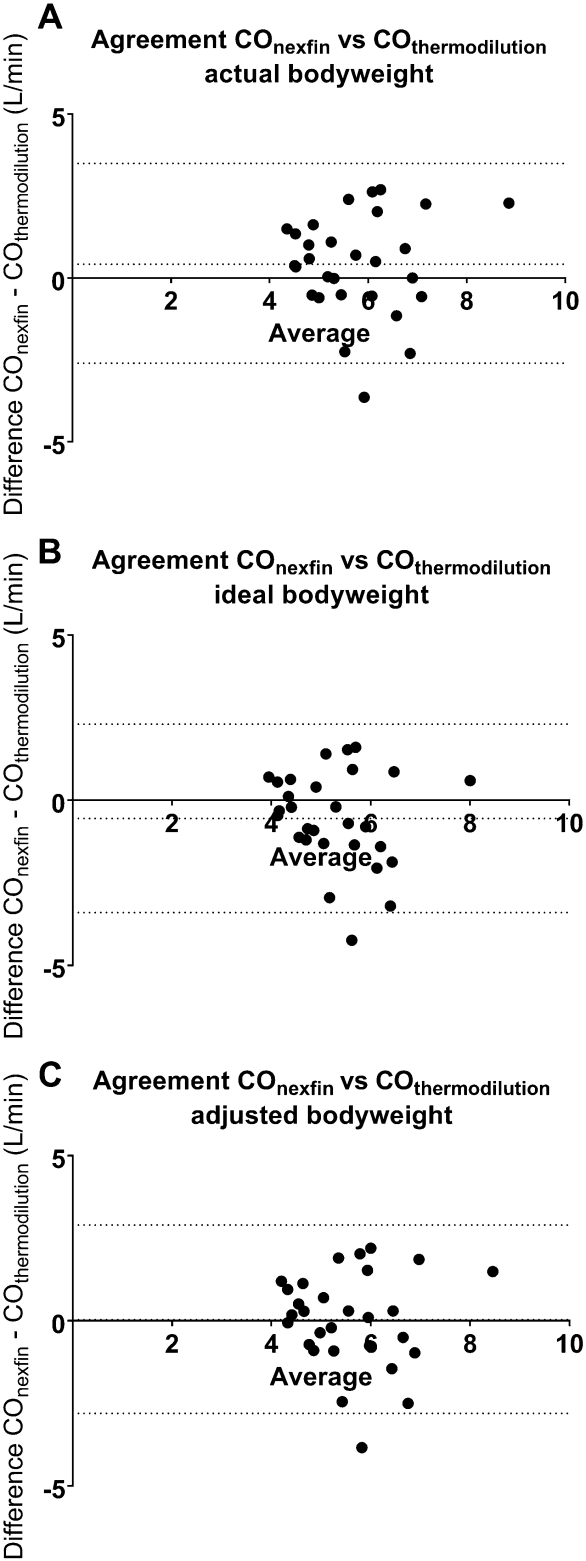
Bland Altman analysis of agreement between Nexfin-derived and thermodilution cardiac output using bodyweight and bodyweight derivatives ideal- and adjusted bodyweight as input to the Nexfin method.** a** Agreement between methods using actual bodyweight, bias 0.42 L min with LOA from −2.6 t 3.5 L min.** b** Agreement between methods using ideal bodyweight, bias −0.55 L min with LOA from −3.4 to 2.3 L min.** c** Agreement between methods using adjusted bodyweight, bias 0.04 L min with LOA from −2.8 to 2.9 L min

Bland Altman analysis of agreement between Nexfin and thermodilution-derived cardiac output values revealed a bias of 0.42 ± 1.55 with limits of agreement (LOA) ranging from −2.6 to 3.5 L min when actual bodyweight was used as input (Fig. 2a). When ideal bodyweight was used as input, the bias was −0.6 ± 1.4 and LOA ranged from −3.4 to 2.3 L min (Fig. 2b). Finally, using adjusted bodyweight as input, bias was 0.04 ± 1.4 and LOA from −2.8 to 2.9 L min (Fig. 2c).

## Discussion

The present study investigated whether adjustment of actual body weight of morbidly obese patients to ideal or adjusted body weight influences the low level of agreement between Nexfin and thermodilution-derived cardiac output measurements. The present study shows that the difference between the Nexfin- and thermodilution-derived cardiac output was moderately correlated with actual bodyweight, suggesting that the bias changes with higher weight. Moreover, our findings show that changing actual body weight to ideal body weight in the Nexfin CO-trek algorithm resulted in worsening of the level of agreement between the Nexfin- and thermodilution-derived cardiac output measurements. In contrast, the use of an adjusted body weight in the Nexfin CO-trek algorithm reduced the bias between non-invasive and invasive cardiac output measurements. Our study shows that the agreement of the Nexfin-derived with invasive cardiac output measurements in morbidly obese patients is influenced by body weight, suggesting that Nexfin-derived cardiac output measurements in patients with a BMI above 35 kg/m^2^ should be interpreted with caution.

Intensified hemodynamic monitoring is important in morbidly obese patients, as the combination of impaired cardiac performance and perioperative stressors may lead to hemodynamic instability and predisposes to complications and increased mortality [1.2]. Invasive measurements with intra-arterial lines and/or thermodilution methods are less favorable in this population, especially during bariatric surgery. Therefore, noninvasive devices such as the Nexfin may be an attractive option to provide information on cardiac function and hemodynamics during surgery.

Cardiac output is higher in obese patients, with a 0.08 L min increase for every kg/m^2^ increase in BMI [17]. In agreement with our findings, thermodilution-derived cardiac output measurements show a positive association between bodyweight and cardiac output. Interestingly, this association was not present when cardiac output was determined by Nexfin, and our findings suggest that Nexfin overestimates cardiac output in the relatively lower range of overweight (100–140 kg) and underestimates cardiac output above this range.

Pulse-contour analysis is used in devices such as the Nexfin to calculate stroke volume and thus cardiac output from the arterial pressure waveform. Although blood pressure measurements by this device seem reliable in the perioperative setting [18, 19] studies show variable results regarding agreement of cardiac output with thermodilution and trending capability [12, 20–23]. In the morbidly obese population, other devices using pulse contour analysis to estimate cardiac output have shown to be unreliable compared to thermodilution [24, 25], and our group demonstrated unacceptable agreement with thermodilution and insufficient trending in morbidly obese patients undergoing bariatric surgery [8].

As described above, the CO-trek algorithm calculates stroke volume by dividing the pulsatile systolic area of the arterial pressure waveform by left ventricular input impedance during ejection. It is based on the Modelflow method which simulates a three-element windkessel model, taking into account aortic impedance, compliance and peripheral resistance [9, 13]. An approximation of impedance is made based on data on aortic compliance from early in vitro studies on aortic samples by Langewouters [10] which describe the non-linear relationships of the aorta. Importantly, these relations are influenced by patients’ age, gender, height and weight, therefore input of patient demographics is required in the Nexfin device. The aortas used were isolated from subjects within a limited range of bodyweights, and it is unknown whether these relations can be extrapolated to the morbidly obese population. Vascular compliance is altered in obesity as these patients have abnormal arterial compliance and vascular tone [26, 27].

We conclude that the agreement of the Nexfin-derived with thermodilution cardiac output measurements in morbidly obese patients is influenced by body weight, and suggest that this is due to the vascular alterations in these patients which hinder application of the CO-trek algorithm used by Nexfin. The improved level of agreement with thermodilution cardiac output when adjusted bodyweight is used as input to the Nexfin suggests directions for improvement of the applicability of this device in the morbidly obese population.
